# The Effect of Inhibiting the Wingless/Integrated (WNT) Signaling Pathway on the Early Embryonic Disc Cell Culture in Chickens

**DOI:** 10.3390/ani14091382

**Published:** 2024-05-04

**Authors:** Wenjie Ren, Dan Zheng, Guangzheng Liu, Gaoyuan Wu, Yixiu Peng, Jun Wu, Kai Jin, Qisheng Zuo, Yani Zhang, Guohui Li, Wei Han, Xiang-Shun Cui, Guohong Chen, Bichun Li, Ying-Jie Niu

**Affiliations:** 1 Joint International Research Laboratory of Agriculture and Agri-Product Safety of the Ministry of Education of China, Yangzhou University, Yangzhou 225009, China; 2 Key Laboratory of Animal Breeding Reproduction and Molecular Design for Jiangsu Province, College of Animal Science and Technology, Yangzhou University, Yangzhou 225009, China; 3 Poultry Institute, Chinese Academy of Agricultural Sciences, Yangzhou 225125, China; 4 Department of Animal Science, Chungbuk National University, Cheongju 28644, Republic of Korea

**Keywords:** chicken, PSC, derivation, small molecules

## Abstract

**Simple Summary:**

In this study, we aimed to improve the culture system for chicken embryonic-derived pluripotent stem cells (PSCs). These cells have immense potential in various fields, such as growth and development studies, vaccine production, and preserving genetic resources. However, establishing a stable and efficient culture system for chicken PSCs remains a challenge. We investigated the effects of different signaling pathways and feeder layers on the derivation and maintenance of chicken PSCs. Our results show that using STO cells as feeder layers, along with specific signaling pathway inhibitors, allows for the efficient derivation of chicken PSC-like cells. These cells exhibit characteristics of pluripotency and can differentiate into various cell types. This research enhances our understanding of chicken PSC culture conditions and lays the groundwork for their biomedical and biotechnological applications.

**Abstract:**

The utilization of chicken embryonic-derived pluripotent stem cell (PSC) lines is crucial in various fields, including growth and development, vaccine and protein production, and germplasm resource protection. However, the research foundation for chicken PSCs is relatively weak, and there are still challenges in establishing a stable and efficient PSC culture system. Therefore, this study aims to investigate the effects of the FGF2/ERK and WNT/β-catenin signaling pathways, as well as different feeder layers, on the derivation and maintenance of chicken embryonic-derived PSCs. The results of this study demonstrate that the use of STO cells as feeder layers, along with the addition of FGF2, IWR-1, and XAV-939 (FIX), allows for the efficient derivation of chicken PSC-like cells. Under the FIX culture conditions, chicken PSCs express key pluripotency genes, such as *POUV*, *SOX2*, and *NANOG*, as well as specific proteins SSEA-1, C-KIT, and SOX2, indicating their pluripotent nature. Additionally, the embryoid body experiment confirms that these PSC-like cells can differentiate into cells of three germ layers in vitro, highlighting their potential for multilineage differentiation. Furthermore, this study reveals that chicken Eyal–Giladi and Kochav stage X blastodermal cells express genes related to the primed state of PSCs, and the FIX culture system established in this research maintains the expression of these genes in vitro. These findings contribute significantly to the understanding and optimization of chicken PSC culture conditions and provide a foundation for further exploration of the biomedical research and biotechnological applications of chicken PSCs.

## 1. Introduction

Pluripotent stem cells (PSCs), a population of cells with self-renewal and pluripotent differentiation capabilities [1], have been widely applied in various fields, such as tissue engineering and regenerative medicine, drug screening and toxicity testing, and gene function research [2,3]. Over the past few decades, stable culture systems for PSCs have only been established in a few species, such as mice and humans [4,5]. Due to the unique genetic characteristics of different species, there are significant differences in the culture systems for PSCs [6,7,8]. Until now, a stable culture system similar to mouse embryonic stem cells (ESCs) has not been established in chickens, and long-term cultured chicken ESCs cannot maintain their germ line transmission ability [9,10,11].

Studies on PSCs in mice and other animals have shown that pluripotency is a temporary and dynamically changing state that can be categorized into naïve, primed, and formative states [12,13,14]. Cells in these states originate from different periods and locations during embryonic development, and culture conditions have been established to maintain these different states’ pluripotency. Mouse naïve, formative, and primed state pluripotent cells are derived from inner cell mass (ICM)/epiblasts at E3.5–4, E5–6, and E7.5, respectively, and the established in vitro cell lines are called ESCs, formative PSCs (fPSCs), and epiblast stem cells (EpiSCs), respectively [15]. These PSCs in different states exhibit significant differences in molecular characteristics, signal dependence, colony morphology, cloning efficiency, metabolic requirements, and epigenetic characteristics [12,16]. In chickens, when the embryo develops to the Eyal–Giladi and Kochav stage X (EGK.X), blastodermal cells (BCs) can form somatic and germ cell chimeras after injection into the sub-blastocoel cavity of other recipient embryos [17], indicating their developmental potential similar to the naïve state of the mouse ICM. Many studies have attempted to establish culture systems for chicken ESCs, aiming to derive cells similar to naïve state ESCs [9,10,11,18,19,20,21]. However, so far, long-term cultured chicken BCs can form somatic chimeras, demonstrating their potential to develop into various tissues and organs, but they cannot produce progeny, indicating the loss of germ line transmission ability. Moreover, different culture systems used in various studies result in different clone and cell morphological characteristics, molecular properties, and signal dependence, with little exploration of metabolic requirements and epigenetic characteristics [9,10,11,18,19,20,21]. These studies have evaluated various culture media, feeder layers, conditioned media, growth factors, serum, serum substitutes, and small molecules for chicken ESC culture, but a highly efficient and widely applicable culture system for chicken PSCs has not been identified for derivation.

In mouse PSC research, FGF2, BMP4, LIF, WNT, TGFβ, and other signaling pathways are involved in the maintenance and differentiation processes of PSCs [12,13]. Particularly, in naïve and primed state PSCs, these signaling pathways often play different or even opposing roles. In the culture of chicken BCs, there have been many studies on FGF2 and LIF, and several studies have shown that adding FGF2 and LIF simultaneously can promote the maintenance of chicken PSCs. However, research on WNT, BMP4, and TGFβ signaling pathways is limited. In the previous research by our laboratory, it was found that two WNT inhibitors, IWR-1 and XAV-939, can prolong the formation and maintenance of primed-like chicken PSCs, but their effects and molecular mechanisms in the derivation and maintenance of chicken PSCs are still not clear. It has been found that the WNT signaling pathway mainly plays a role in maintaining the pluripotent state of naïve and primed PSCs [22], while it needs to be inhibited in mouse region-selective epiblast stem cells (rsEpiSCs) [23]. However, whether chicken BCs can generate stem cells under conditions of WNT pathway inhibition has not been tested.

This study aims to investigate the effects of FGF2 and WNT signaling pathway inhibitors (IWR-1 and XAV-939) in culturing chicken BCs. The findings of this study indicate that the concurrent addition of FGF2, IWR-1, and XAV-939, along with the use of STO feeder cells (FIX system), can effectively produce PSCs exhibiting a morphology similar to the primed state. The identification of these putative stem cells reveals that after being cultured in the FIX system, they still expressed pluripotency genes and proteins. Additionally, the embryoid body (EB) formation experiment demonstrates their potential to differentiate into cells of three germ layers. Furthermore, chicken BCs express primed-state PSC-related genes, and the FIX system maintains the expression of these genes. The findings of this study lay the foundation for further understanding the developmental mechanisms of chicken pluripotent cells and the establishment of a comprehensive culture system for chicken PSCs.

## 2. Materials and Methods

All chemicals and reagents utilized in this study were sourced from GIBCO BRL (Grand Island, NY, USA) unless specified otherwise. 

### 2.1. Ethics Statement

The experimental procedures involving animal subjects were rigorously reviewed and approved by the Experimental Animal Ethics Committee of Yangzhou University. Fertilized chicken eggs utilized in this study were obtained from the Jiangsu Institute of Poultry Sciences under strict adherence to ethical guidelines and regulations governing animal research. The ICR mice were kept under controlled conditions of temperature (20–23 °C) and lighting (12 h light–dark cycle) and had ad libitum access to food and water. Pregnant mice at 12.5 days were used for embryo collection, which were then used for the isolation and culture of mouse embryonic fibroblasts (MEF).

### 2.2. Small Molecules and Cytokines 

The following small molecules and cytokines were used in this study: dimethyl sulfoxide (DMSO, Sigma-Aldrich, Saint Louis, MO, USA) as a solvent, FGF2 (MCE, 4 ng/mL), IWR-1 (MCE, 2 μM), XAV-939 (MCE, 2.5 μM), CHIR-99021 (MCE, 0.2 μM), and PD0325901 (MCE, 1 μM).

### 2.3. Cell Culture Media

Avian KOSR medium was composed of KO-DMEM, 15% KnockOut serum replacement, 0.2% chicken serum (Bioland Scientific, LLC., Paramount, CA, USA), 1× B-27 supplement, 1× N-2 supplement, 1× GlutaMax, 0.1 mM β-mercaptoethanol (Sigma-Aldrich, MO, USA), 1× EmbryoMax nucleosides (Millipore, Louis Missouri, MA, USA), 1× NEAA, 1 mM sodium pyruvate, and 1× penicillin/streptomycin (Solarbio, Beijing, China).

FIX medium contained Avian KOSR medium as the basal medium and 4.0 ng/mL of human FGF2 (MCE), and 2 μM of IWR-1 (MCE) and 2.5 μM of XAV-939 (MCE) were added to the medium.

### 2.4. Preparation of Feeder Layers

To obtain MEF, mouse embryos were dissected from 12.5-day pregnant mice under sterile conditions. The embryos were washed twice with PBS to remove the limbs, head, tail, and viscera. After PBS washing, the remaining tissue was minced and digested with trypsin-EDTA to form a cell suspension, which was then seeded in culture flasks for cultivation with a feeder medium (DMEM containing 10% fetal bovine serum and 1× penicillin/streptomycin). MEF at passages 2–3 were used for feeder layer preparation. STO (S, SIM; T, 6-thioguanine resistant; O, ouabain resistant) cells were purchased from ATCC (CRL-1821) and cultured with a feeder medium. To prepare the feeder layers, the prepared MEF and STO cells were treated with 10 μg/mL of mitomycin C for 2 h and then frozen in liquid nitrogen for later use. On the day before using the feeder layers, the thawed inactivated MEF or STO cells were seeded onto culture dishes coated with 0.1% gelatin.

### 2.5. PSCs Isolation and Culture

Freshly fertilized eggs of Rugao yellow chickens were collected and disinfected with 70% ethanol. The BCs from the area pellucida of the EGK.X stage embryos were isolated as previously described. Briefly, the eggshells were cracked open with forceps, exposing the embryo. A sterilized and dried filter paper ring was placed on the embryo, and then the embryo was separated from the yolk by cutting along the outer edge of the ring with scissors. The embryo was lifted to separate it from the yolk, washed with PBS to remove egg yolk, and BCs in the area pellucida were isolated using a needle under a microscope. The BCs were dissociated through gentle pipetting and then centrifuged at 1500 rpm for 5 min to pellet the cells. BCs from each embryo were plated on mitotically inactivated feeder cells. The culture medium was changed daily, and after 2–3 days, PSC-like colonies were dissociated with Accutase (40506ES60, Yeasen, Shanghai, China) for 5–7 min. The colonies were then plated on a 12-well dish that was seeded with mitomycin C-treated MEF or STO cells.

### 2.6. Formation and In Vitro Differentiation of EBs

After dissociating the PSCs cultured up to the fourth generation with Accutase, the cells were collected in a 15 mL centrifuge tube and centrifuged at 1000 rpm for 5 min, and the supernatant was discarded. The collected cells were plated in a culture dish and allowed to adhere for 1 h. Then, the PSCs in the supernatant were collected (this process eliminated feeder cells), centrifuged at 1000 rpm for 5 min, and resuspended in an avian KOSR medium. The cells were then plated in ultra-low-attachment multi-well plates (CLS3473, Corning, NY, USA) and cultured in a 37 °C, 5% CO_2_ incubator for 5 days. Subsequently, to assess the ability of PSCs to differentiate into cells representing the three germ layers, the formed EBs were plated on a 1% gelatin-coated culture dish and cultured for 5 days in a 37 °C, 5% CO_2_ incubator to observe their differentiation ability. Following incubation, the cells were subjected to immunofluorescence staining using antibodies against PAX6, AFP, and smooth muscle actin.

### 2.7. RNA Isolation and Reverse Transcription

Total RNA extraction from PSCs was performed using Trizol reagent (Vazyme, Nanjing, China) according to the manufacturer’s instructions. Briefly, one million PSCs were collected and lysed in Trizol reagent, followed by chloroform extraction and isopropanol precipitation to isolate RNA. The RNA pellet was washed with ethanol, air-dried, and resuspended in DNase-/RNase-free distilled water. The concentration and purity of the isolated RNA were determined spectrophotometrically using a NanoDrop 2000 spectrophotometer (Thermo Scientific™, Waltham, MA, USA). Subsequently, reverse transcription of RNA to complementary DNA (cDNA) was carried out using the HiScript^®^ III RT SuperMix (+gDNA wiper) kit (Vazyme, Nanjing, China), as per the manufacturer’s protocol. 

### 2.8. Polymerase Chain Reaction (PCR) and Agarose Gel Electrophoresis

PCR amplification of target genes was performed using PrimeSTAR^®^ Max DNA polymerase (Takara Bio Inc., Kyoto, Japan) according to the manufacturer’s instructions. The PCR reaction mixture containing template DNA, primers (Table 1), and PCR master mix was subjected to thermal cycling conditions consisting of an initial denaturation step at 98 °C for 5 min, followed by 35 cycles of denaturation at 98 °C for 10 s, annealing at 60 °C for 15 s, and extension at 72 °C for 30 s. A final extension step at 72 °C for 5 min was performed before holding the samples at 4 °C. The PCR products were then separated using agarose gel electrophoresis on a 1.5% agarose gel containing RedSafe nucleic acid staining solution (INtRON biotechnology, Gyeonggi, South Korea) and visualized under UV illumination. 

### 2.9. Quantitative Real-Time PCR (qRT-PCR)

The qRT-PCR analysis was executed utilizing the ChamQ Universal SYBR qPCR Master Mix kit (Vazyme, Nanjing, China) in strict accordance with the manufacturer’s guidelines. In brief, the qPCR reaction mixture, comprising cDNA, gene-specific primers (as listed in Table 1), and master mix, underwent a series of precisely controlled thermal cycling conditions. This included an initial denaturation step at 95 °C for 30 s, followed by 40 cycles of denaturation at 95 °C for 10 s, annealing at 60 °C for 30 s, and extension at 72 °C for 15 s. Subsequently, a final extension step at 72 °C for 5 min was conducted before holding the samples at 4 °C. The expression levels of the target genes, namely *POUV*, *SOX2*, *NANOG*, *AXIN1*, *AXIN2*, *FGF5*, *OTX2*, *PAX6*, *HNF1A*, and *PPARA*, were quantified utilizing the 2^−ΔΔCT^ method [26], with normalization to the expression levels of the reference gene GAPDH.

### 2.10. Immunofluorescence

Immunofluorescence staining was conducted to visualize the expression and localization of specific proteins within cells. After washing with PBS, cells were fixed in 4% paraformaldehyde, permeabilized with PBS containing 0.5% Triton X-100, and blocked with blocking solution (PBS containing 1.0% BSA). The cells were then subjected to overnight incubation at 4 °C with primary antibodies targeting proteins of interest, including SSEA-1, EMA-1, C-KIT, SOX2, PAX6, AFP, and smooth muscle actin. Following primary antibody incubation, cells were exposed to appropriate secondary antibodies, namely donkey anti-rabbit IgG or goat anti-mouse IgG, at room temperature. Nuclei were counterstained with DAPI, and the stained cells were mounted with an anti-fluorescence quenching agent and examined under a confocal microscope (Zeiss LSM 710 Meta, Zeiss, Oberkochen, Germany). Image acquisition and analysis were performed using Olympus Fluoview 4.1a viewer (Olympus, Tokyo, Japan).

### 2.11. Statistical Analysis

The transcriptomic data of primed-state genes was derived from publicly available data in the NCBI GEO database (GSE174603) [27]. All experiments were performed in triplicate, and data are presented as mean ± standard error of the mean (SEM). Statistical analysis was conducted using the one-way analysis of variance and Student’s *t*-test to determine significant differences between experimental groups, with *p* < 0.05 considered statistically significant. Prior to analysis, percentage data were subjected to arcsine transformation to meet the assumptions of parametric tests. Statistical calculations were performed using SPSS software v.19 (SPSS, Inc., Chicago, IL, USA).

## 3. Results

### 3.1. FGF2, IWR-1, and XAV-939 Promote Efficient Derivation of Chicken PSC-like Cells from BCs

**Figure 1:**
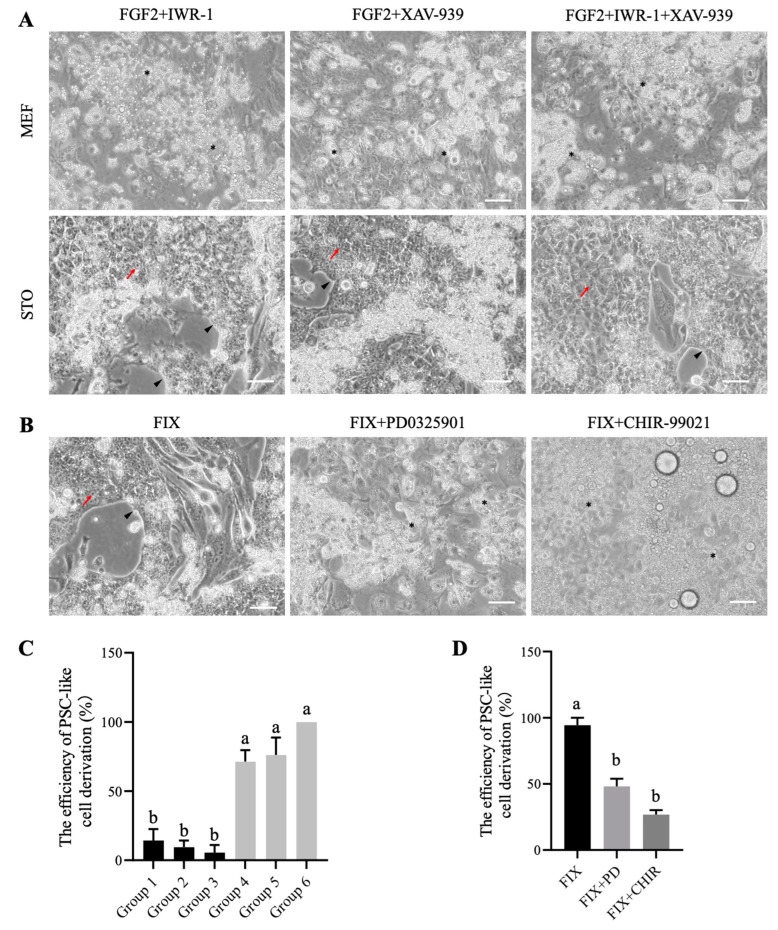
Promotion of efficient derivation of chicken PSC-like cells from BCs by FGF2, IWR-1, and XAV-939. Representative images (**A**) and derivation efficiency (**C**) of clone formation by BCs under different feeder cell conditions (STO/MEF) and combinations of cytokines/small molecules (FGF2 + IWR-1, FGF2 + XAV-939, FGF2 + IWR-1 + XAV-939). (**B**,**D**) Addition of PD0325901 or CHIR-99021 prevented the formation of PSC-like cells from BCs. Scale bar represents 100 µm. The efficiency of PSC-like cell derivation is calculated by dividing the number of embryos capable of PSC-like cell formation by the total number of embryos. Red arrows indicate single PSC-like cells, black triangles mark the edges of clones, and black asterisks (*) denote areas of differentiation. Different lowercase letters indicate significant differences.

Previous studies have shown that the addition of FGF2 and either IWR-1 or XAV-939 (inhibitors of the WNT pathway) promotes the formation of mouse rsEpiSCs with high efficiency [23]. To investigate whether the FGF2 and WNT signaling pathways are involved in the derivation and maintenance of chicken embryonic PSCs, this study examined the effects of adding FGF2, IWR-1, and XAV-939 on the derivation of PSCs. After obtaining chicken EGK.X blastoderms, the area pellucida regions were isolated and cultured individually in wells of a 24-well plate. Based on the different cytokines and inhibitors added to the culture medium, as well as the type of feeder cells used, the experiments were divided into six groups. The results showed that the growth of BCs varied among different groups (Figure 1A,C). In Groups 1–3, using MEF as feeder cells, the addition of FGF2 + IWR-1 (Group 1), FGF2 + XAV-939 (Group 2), or FGF2 + IWR-1 + XAV-939 (Group 3) resulted in the differentiation of the majority of BCs cells, with only 5.56%–14.29% of the wells containing PSC-like cells. However, when STO feeder cells were combined with FGF2 + IWR-1 (Group 4), FGF2 + XAV-939 (Group 5), or FGF2 + IWR-1 + XAV-939 (Group 6), the formation efficiencies significantly increased to 71.4%, 76.2%, and 100%, respectively (Figure 1C). The formed clones resemble those reported by van de Lavoir [9], exhibiting characteristics such as large nuclei, prominent nucleoli, and flattened morphology. Because the combination of STO feeder cells and FGF2 + IWR-1 + XAV-939 (FIX system) displayed the highest efficiency in PSC formation, this condition was chosen for subsequent experiments.

To verify that the addition of FGF2 + IWR-1 + XAV-939 indeed maintains the formation of PSC-like cells by activating the FGF2/ERK signaling pathway and inhibiting the Wnt/β-catenin signaling pathway, the ERK inhibitor PD0325901 and the GSK-3β inhibitor CHIR-99021 (which activates the Wnt signaling pathway) were added separately to test their effects on the FIX system-derived PSC-like cells. The results indicated that the addition of PD0325901 or CHIR-99021 significantly reduced the formation of PSC-like cells from BCs (Figure 1B,D), indicating the importance of the FGF2/ERK and Wnt/β-catenin signaling pathways in maintaining pluripotency.

### 3.2. Effect of Addition of Cytokines or Small Molecules on Maintenance of Chicken PSCs in FIX System

**Figure 2:**
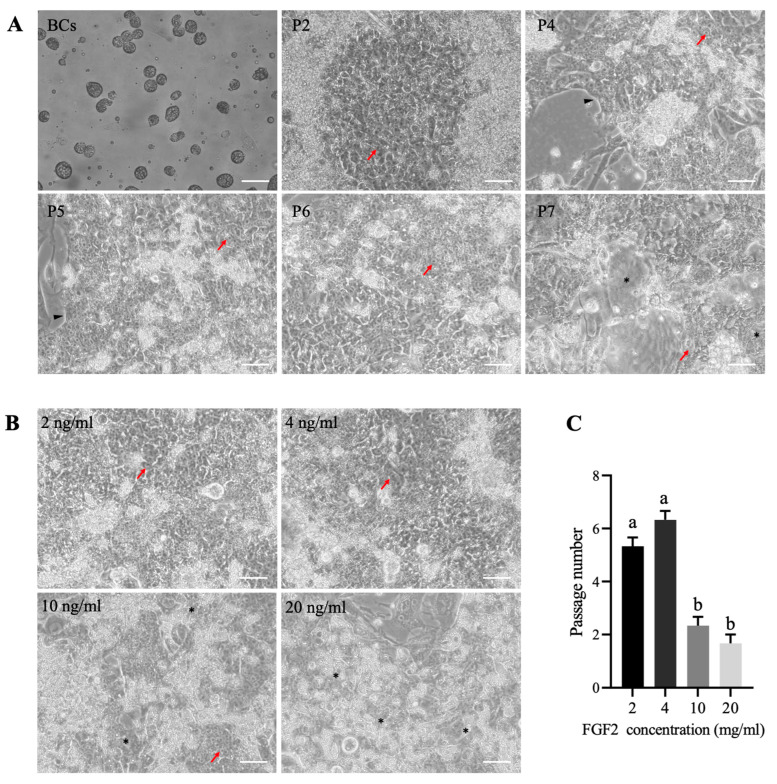
Passage ability of PSC-like cells in FIX medium. (**A**) Representative images of BCs and their derived PSC-like cells during the passage process. P1–P7 are referring to passages 1–7. (**B**,**C**) Effects of different concentrations of FGF2 in FIX medium on the passage ability of PSC-like cells. Scale bar represents 100 µm. Red arrows indicate single PSC-like cells, black triangles mark the edges of clones, and black asterisks (*) denote areas of differentiation. Different lowercase letters indicate significant differences.

Although the FIX system can efficiently derive chicken PSC-like cells, it is still unknown whether it can support the long-term propagation of PSC-like cells. Therefore, we performed passaging of the derived PSC-like cells and found that the cells could pass up to the sixth generation. However, beyond the sixth generation, cell proliferation gradually slowed down, and the cells began to lose their original nuclei, indicating the onset of differentiation. (Figure 2A).

Previous studies have shown that the concentration of FGF2 is crucial for maintaining primed PSCs. While FGF2 at 4 ng/mL can derive primed PSCs, 20 ng/mL is required for efficient maintenance of primed PSCs during long-term passaging [28]. Therefore, to investigate the effects of different concentrations of FGF2 on the derivation and maintenance of chicken PSCs, we added 2 ng/mL, 4 ng/mL, 10 ng/mL, and 20 ng/mL of FGF2 along with IWR-1 + XAV-939 and observed their impact on the passaging ability of chicken PSC-like cells. We observed that there was no significant difference in the passaging ability of PSCs between the 2 ng/mL and 4 ng/mL FGF2 groups (Figure 2B,C). However, increasing the concentration of FGF2 did not enhance the passaging ability of chicken PSCs. On the contrary, the addition of 10 ng/mL and 20 ng/mL of FGF2 significantly reduced the passaging ability of chicken PSCs (Figure 2B,C). Therefore, subsequent experiments were conducted using 4 ng/mL of FGF2.

### 3.3. Detection of Pluripotency in PSC-like Cells Derived from the FIX System

**Figure 3:**
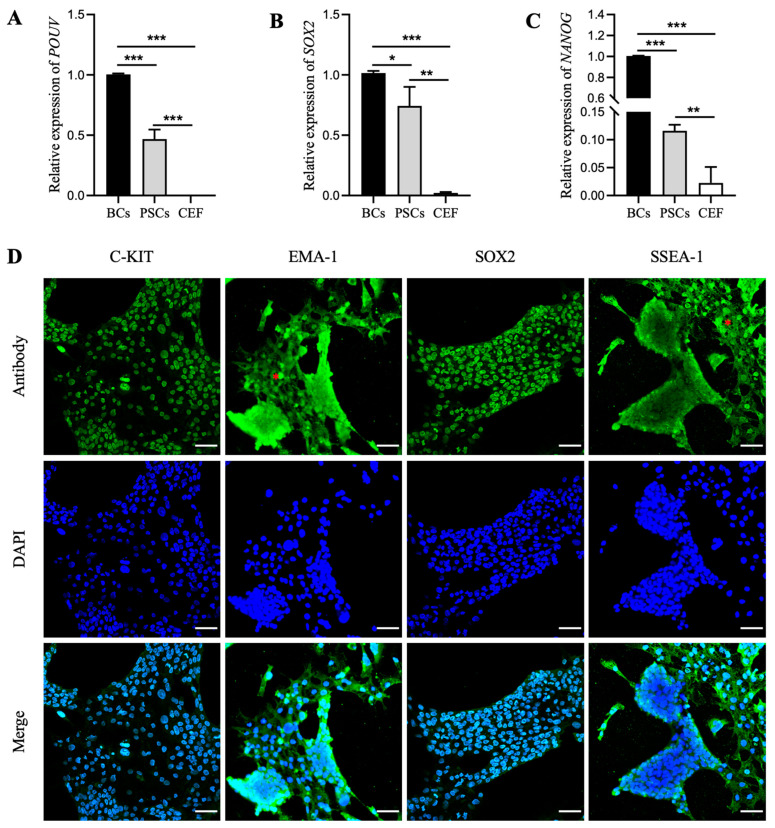
Detection of pluripotency in PSC-like cells derived from the FIX system. The qRT-PCR analysis of *POUV* (**A**), *SOX2* (**B**), and *NANOG* (**C**) gene expression levels in PSC-like cells compared with BCs and CEF. Blastodermal cells (BCs) and fibroblasts (CEF) were used as positive and negative controls, respectively. * indicates significant differences in gene expression. * indicates *p* < 0.05. ** indicates *p* < 0.01. *** indicates *p* < 0.001. (**D**) Immunofluorescence staining analyses showing expression of C-KIT, EMA-1, SOX2, and SSEA-1 proteins in PSC-like cells. The cells marked with a red asterisk (*) indicate potential differentiation. Scale bar represents 40 µm. The PSC-like cells used in this experiment were from the sixth generation.

To assess whether the PSC-like cells obtained from the FIX culture system possess molecular characteristics of PSCs, we conducted an analysis of the expression of pluripotency genes. The qRT-PCR analysis revealed that while the expression levels of *POUV*, *SOX2*, and *NANOG* genes were significantly higher in PSCs compared to CEF, the expression levels of these genes were notably reduced in PSCs compared to BCs cells. Particularly, *NANOG* was approximately 10 times lower in expression in PSCs compared to BCs (Figure 3A–C). This may be a reason why these cells were unable to undergo long-term passaging. Finally, immunofluorescence staining demonstrated that the PSC-like cells derived from the FIX system still expressed PSC-specific proteins, such as C-KIT, EMA-1, SOX2, and SSEA-1 (Figure 3D). These results indicate that the PSC-like cells derived from the FIX system can maintain pluripotency for a certain period.

### 3.4. Expression Analysis of Primed-State Genes in Chicken PSC-like Cells Derived from the FIX System

**Figure 4:**
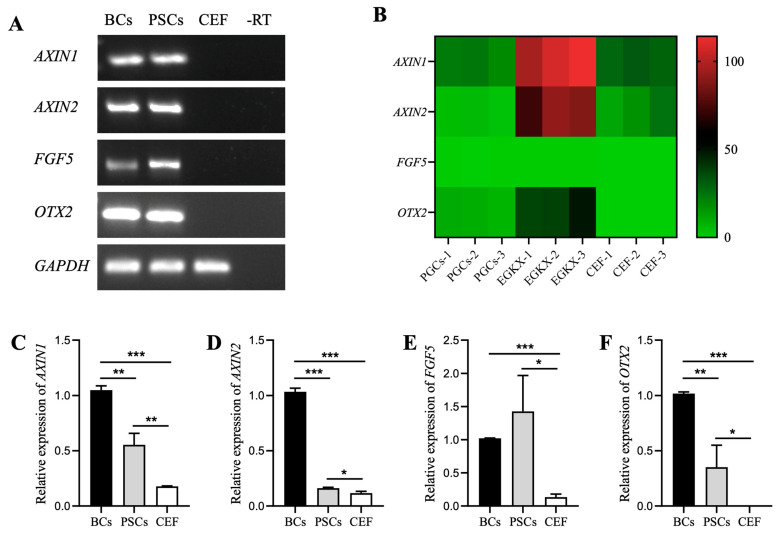
Expression analysis of primed-state genes in chicken PSC-like cells derived from the FIX system. (**A**) RT-PCR and agarose gel electrophoresis analyses demonstrating expression of primed-state genes in PSC-like cells. BCs and CEF were used as positive and negative controls, respectively. (**B**) Heatmap analyses reveal significantly higher levels of primed-state gene expression in BCs when compared to primordial germ cells (PGCs) and CEF. The qRT-PCR analysis of *AXIN1* (**C**), *AXIN2* (**D**), *FGF5* (**E**), and *OTX2* (**F**) gene expression levels in PSC-like cells compared with BCs and CEF. The PSC-like cells used in this experiment were from the sixth generation. * indicates significant differences in gene expression. * indicates *p* < 0.05. ** indicates *p* < 0.01. *** indicates *p* < 0.001.

Studies have shown that the combination of FGF2 and IWR-1 can effectively derive and maintain mouse rsEpiSCs, which exhibit gene expression patterns characteristic of primed-state PSCs [23]. To determine whether the PSC-like cells derived from the FIX system have a primed state, we performed RT-PCR experiments and found that both isolated chicken BCs and PSC-like cells derived from the FIX system expressed genes *AXIN1*, AXIN2, *OTX2*, and *FGF5* [13,23,29] (Figure 4A). To validate this result, transcriptome data [27] analysis revealed that compared to primordial germ cells (PGCs) and chicken embryonic fibroblasts (CEF), EGK.X stage cells indeed expressed higher levels of *AXIN1*, *AXIN2*, *FGF5*, and *OTX2* genes (Figure 4B), further demonstrating the presence of cells in blastoderm at the EGK.X stage expressing primed state-related genes, which were maintained during culturing in the FIX system. The qRT-PCR analysis also showed that compared to the BC group, the PSC-like cells derived from the FIX system expressed lower levels of *AXIN1*, *AXIN2*, *FGF5*, and *OTX2* genes (Figure 4C–F). However, these genes were significantly higher in expression compared to CEF cells. These results suggest that the FIX system can partially maintain the expression of primed-state genes.

### 3.5. PSCs Derived from the FIX System Have the Potential to Differentiate into Three Germ Layer Cells

**Figure 5:**
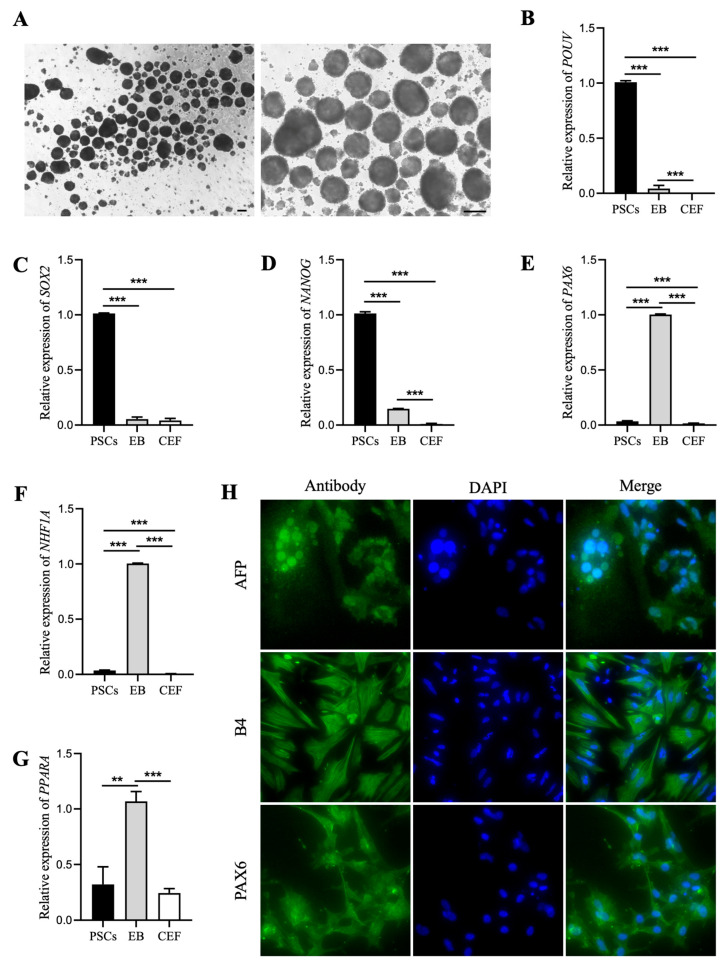
Differentiation potential of PSC-like cells derived from the FIX system into three germ layer cells. (**A**) Images of embryoid bodies formed by PSC-like cells. Scale bar represents 200 µm. The qRT-PCR analysis of *POUV* (**B**), *SOX2* (**C**), *NANOG* (**D**), *PAX6* (**E**), *NHF1A* (**F**), and *PPARA* (**G**) gene expression levels in EBs compared with PSCs. CEF was used as a negative control. ** indicates *p* < 0.01. *** indicates *p* < 0.001. (**H**) Immunofluorescence staining analyses demonstrated the expression of AFP, B4, and PAX6 proteins in the differentiated cells derived from PSC-like cells. PAX6 is a marker for ectoderm, AFP is a marker for endoderm, and smooth muscle actin (B4) is a marker for mesoderm. Scale bar represents 40 µm. The PSC-like cells used in this experiment were from the fourth generation.

To test whether the PSC-like cells derived from the FIX system have the ability to differentiate into multiple lineages, we conducted EB formation experiments. The PSC-like cells were dissociated and plated onto low attachment culture dishes without adding FGF2, IWR-1, and XAV-939, as well as without STO feeder cells, to assess their ability to form EBs. Under inverted microscopy, cystic EBs were observed on the second day of culture, and the EBs further increased in size by the fifth day (Figure 5A). The qRT-PCR results indicated that the pluripotency genes *POUV*, *SOX2*, and *NANOG* were highly expressed in PSCs, but their expression significantly decreased after EB formation (Figure 5B–D). Conversely, the expression levels of *PAX6*, *PPARA*, and *HNF1A* were upregulated after EB formation (Figure 5E–G). Finally, the formed EBs were transferred onto gelatin-coated culture plates, and it was observed that these cells differentiated into various types of somatic cells. Immunofluorescence staining showed that these cells expressed proteins such as AFP, B4, and PAX6 (Figure 5H). These results indicate that PSC-like cells derived from the FIX system have the ability to differentiate into three germ layer cells.

## 4. Discussion

PSCs exhibit the remarkable capabilities of self-renewal and the ability to generate somatic cell and germline chimeras without the introduction of any external genes. They have important application prospects and play a crucial role in the conservation and restoration of animal germplasm resources [30]. Therefore, the development of PSC culture systems has always been a hot research topic. In this study, a chicken PSC culture system was established through systematic exploration, in which FGF2 + IWR-1 + XAV-939 were added and STO was used as the feeder layer. Within this system, isolated chicken BCs efficiently formed primed-state clones. The identification of the generated clones confirmed their sustained expression of pluripotency genes and ability to form three germ layers.

Different animals have slightly different PSC culture systems due to interspecies differences [6,7,8]. As an important agricultural animal and model organism, establishing a culture system for chicken PSCs is of great significance. Currently, many culture systems have been attempted in chickens. Among them, Pain and his colleagues established a long-term in vitro culture system for chicken ESCs using STO feeder cells and the addition of IGF1, mSCF, hIL-6, and hIL6-sR to the culture medium [11]. Petitte derived and cultured chicken ESCs long-term using Buffalo rat liver-conditioned medium (BRL-CM) and STO feeders [10]. Recently, Zhang and his colleagues described a method for culturing pluripotent blastodermal cells in vitro with the support of DF-1 feeder and hbFGF, mSCF, and hLIF [20]. In addition, the addition of PD0325901, SB431542, and LIF, named R2i + LIF, improved ESC-like cell derivation from stages X- and HH28 embryos [19]. However, so far, these culture systems are limited to a few laboratories, and a reproducible and widely applicable culture system has not been established, as in mice or humans. This also suggests that the exact regulatory mechanisms involved in the development of chicken embryonic pluripotent cells remain unclear. To explore the roles of FGF2/ERK and Wnt/β-catenin signaling in the proliferation of chicken BCs and the maintenance of pluripotency, this study activated the FGF2/ERK signaling pathway by adding FGF2 and suppressed the Wnt/β-catenin signaling pathway by adding IWR-1 and XAV-939. The results showed that in this system, with STO as the feeder cells, PSCs could be derived very efficiently. However, when PD0325901 or CHIR-99021 were used to inhibit FGF2/ERK or activate Wnt/β-catenin signaling pathways, the effects of FGF2 or IWR-1 and XAV-939 were counteracted, and the cells quickly differentiated. This indicates that activating the FGF2/ERK signaling pathway and inhibiting the Wnt/β-catenin signaling pathway are both crucial for BC proliferation and pluripotency maintenance. When MEF was used as the feeder layer, the effects of FGF2, IWR-1, and XAV-939 were reduced to some extent, suggesting that the STO feeder cells are also crucial for maintaining BC proliferation and pluripotency. Therefore, by combining the use of STO as the feeder layer cells and adding FGF2, IWR-1, and XAV-939, a short-term proliferation culture system for chicken BCs was established. To further optimize the FIX system and prolong the in vitro passage of chicken BCs, we tested various concentrations of FGF2. However, none of the concentrations resulted in an increased passage number of BCs. Therefore, additional experimentation with other cytokines or small molecules, or the optimization of the FIX system through the combination of different substances, may be necessary [31,32].

Pluripotency is a crucial characteristic of PSCs [22,33]. In the current study, we conducted several tests to investigate whether chicken BCs can retain pluripotency after being cultured in the FIX system. These tests included examining the morphology of cells and clones, evaluating the expression of pluripotency genes and proteins in the obtained PSC-like cells [11,19]. Our results showed that BCs cultured in the FIX system showed high expression of *POUV*, *SOX2*, and *NANOG*, which is consistent with previous research. Additionally, immunofluorescence staining demonstrated specific protein staining for C-KIT, EMA-1, SOX2, and SSEA-1 in these cells, further confirming their pluripotent characteristics. However, it is noteworthy that in comparison to BCs, the FIX system exhibited significantly reduced expression levels of *POUV*, *SOX2*, and *NANOG*. Additionally, immunofluorescence staining also revealed potential differentiation in some cells. This may explain why BCs cannot be passaged for long periods in this system, and further investigation is needed to find appropriate solutions. To determine if chicken BCs still have the potential for multi-directional differentiation after culture in the FIX system, we conducted EB experiments. We found that PSC-like cells derived from the FIX system effectively formed EBs when FGF2, IWR-1, XAV-939, and STO feeder cells were removed. During the formation of EBs, the expression levels of pluripotency genes *POUV*, *SOX2*, and *NANOG* were significantly decreased, while the expression levels of germ layer-related genes *PAX6*, *PPARA*, and *HNF1A* were significantly increased. Immunofluorescence staining of differentiated cells further confirmed their differentiation into AFP-, B4-, and PAX6-positive cells. Overall, this experiment further emphasized the importance of FGF2, IWR-1, XAV-939, and STO feeder cells in maintaining the pluripotency of chicken BCs.

PSCs can be categorized into naïve, primed, and formative states based on their developmental potential and distinct cellular characteristics [12,13,14]. However, there is limited research on the developmental state of PSCs in chickens. Jean et al. discovered, through transcriptome analysis, that ESCs cultured in the system established by Pain bear more resemblance to naïve-state ESCs in mice [34]. Unfortunately, ESCs cultured in this system are not capable of transmitting germ cells like mouse ESCs [11]. The specific reasons for this limitation require further investigation. The three factors utilized in this study are pivotal for deriving and maintaining primed-state mouse rsEpiSCs [23]. Consequently, the expression levels of key genes associated with the primed state, such as *AXIN1*, *AXIN2*, *FGF5*, and *OTX2*, were examined in BCs and PSC-like cells derived from the FIX system. It was observed that these genes were highly expressed in PSC-like cells. Interestingly, prior to culture, these genes were also highly expressed in BCs. To validate the expression of primed state-related genes in BCs, transcriptome data [27] were analyzed, and the findings confirmed that these genes were indeed highly expressed in EGK.X cells. However, the expression levels of primed state-related genes were significantly lower compared to EGK.X cells. The most plausible explanation is that EGK.X cells already possess characteristics of the primed state, and the FIX system partially sustains the primed state, thereby facilitating the proliferation and maintenance of chicken BCs. Therefore, further investigation is needed to explore methods for sustaining the continuous expression of primed state-related genes.

## 5. Conclusions

In conclusion, this study utilized N2B27 and KSR as replacements for serum, employed STO cells as feeder layer cells, and added FGF2, IWR-1, and XAV-939 to the culture medium to effectively generate clones with distinct nucleoli and flattened morphology resembling primed-state PSCs. However, the specific role of the components within the STO feeder layer cells remains unclear, while the basic culture medium, growth factors, additives, etc., were all commercially available, providing convenience for the future use of this system in different laboratories. The newly developed culture system proves to be suitable for the short-term culture of PSCs, with high efficiency in generating PSC-like cells. However, it is not optimal for the long-term proliferation and maintenance of PSCs, thus requiring further optimization to ensure the desired effects during PSC maintenance and derivation.

## Figures and Tables

**Table 1:** The primers used in the present study.

Gene	Primer Sequence	Annealing Temp.	Product Size (bp)	Reference
*POUV* F	GTTGTCCGGGTCTGGTTCT	60 °C	189	[24]
*POUV* R	GTGGAAAGGTGGCATGTAGAC			
*SOX2* F	GTGAACCAGAGGATGGACAGTTACG	60 °C	185	[25]
*SOX2* R	TGCGAGCTGGTCATGGAGTTG			
*NANOG* F	GGTTTCAGAACCAACGGATG	60 °C	121	[24]
*NANOG* R	GTGGGGGGTCATATCCAGGTA			
*AXIN1* F	CGGATACGATGTCGCTCACA	60 °C	108	—
*AXIN1* R	CACGTCCATTTGCTTTGGCA			
*AXIN2* F	AAGCAAAATGCTGCCTTCGG	60 °C	161	—
*AXIN2* R	CTGGGGCAAAGACATAGCCA			
*FGF5* F	GGGGATCGTAGGAATCCGAG	60 °C	197	—
*FGF5* R	TGTTGAGGGCCACATACCAC			
*OTX2* F	GGATTTGTTGCATCCGTCCG	60 °C	194	—
*OTX2* R	TGAACCACACCTGCACTCTG			
*PAX6* F	GAGAACCCACTATCCCGATGT	60 °C	200	—
*PAX6* R	GGTAAACGCTTGTGCTGAAAC			
*HNF1A* F	AGCCAGAACCTACTGAGCAC	60 °C	288	—
*HNF1A* R	GCTCCCCATGCTGTTTATCAC			
*PPARA* F	AATCACCCAGTGGAGCAGAAA	60 °C	266	—
*PPARA* R	CTCAGACCTTGGCATTCGTC			
*GAPDH* F	GAGGGTAGTGAAGGCTGCTG	60 °C	113	[24]
*GAPDH* R	CATCAAAGGTGGAGGAATGG			

## Data Availability

The data presented in this study are available on request from the corresponding author due to reasonable request.
